# Comparison of the Effectiveness of Simple Plate Fixation and Plate Combined with Local Fixation of Broken Ends in the Treatment of Oblique Fracture of Midshaft Clavicle

**DOI:** 10.1111/os.13310

**Published:** 2022-05-23

**Authors:** Gong‐ming Gao, Yi Zhang, Hai‐bo Li, Lu‐ming Nong, Xin‐die Zhou, Wei Jiang, Long Han

**Affiliations:** ^1^ Department of Orthopedics The Affiliated Changzhou No.2 People's Hospital of Nanjing Medical University Changzhou China

**Keywords:** Classification, Clavicle, Shoulder joint, Surgical fixation devices, X‐rays

## Abstract

**Objective:**

To compare the clinical efficacy of performing simple plate fixation with that using a plate combined with fracture end fixation to investigate the necessity of fracture end fixation outside the plate in cases of oblique fracture of the middle clavicle.

**Methods:**

This was a retrospective follow‐up study of patients with middle clavicle oblique fractures (Robinson types 2A1 and 2A2) between 2015 and 2020. Patients were divided into two groups according to their treatment options: the simple plate fixation (SPF) group (*n* = 79; 43 men and 36 women; average age, 46.37 ± 14.54 years) and the plate combined with fracture local fixation (PLFP) group (*n* = 81; 36 men and 45 women; average age, 48.42 ± 12.55 years). Intraoperative blood loss, operation time, postoperative fracture healing time, postoperative shoulder function score (Constant–Murley and disabilities of the arm, shoulder, and hand [DASH] scores), clinical complications, and postoperative subjective satisfaction were compared between the two groups.

**Results:**

One hundred sixty patients with a sufficient follow‐up period were included in the final analysis: 79 in the SPF group (follow‐up time: 16.24 ± 3.94 months) and 81 in the PLFP group (follow‐up time: 16.15 ± 3.43 months). Age, sex, body mass index, follow‐up duration, fracture classification, and cause of injury were not significantly different between the two groups. There was no significant difference in blood loss, Constant–Murley and DASH scores, follow‐up period, and postoperative subjective satisfaction between the two groups (*P >* 0.05). The fracture healing time was shorter in the PLFP group than in the SPF group (4.41 ± 0.99 *vs*. 4.87 ± 1.60 months, *P* < 0.05), but the operation duration was longer in the PLFP group than in the SPF group (65.48 ± 16.48 min, *P* < 0.05). There were seven (complication rate, 8.86%) and five (complication rate, 6.17%) cases that had complications in the SPF and PLFP groups, respectively. There was no significant difference in the complication rates between the two groups (*P >* 0.05).

**Conclusion:**

Although the healing time was shorter in the PLFP group than in the SPF group, the clinical efficiency of the two methods in the treatment of oblique fracture of the middle clavicle was similar.

## Graphical abstract

The last half of the picture shows the schematic diagram of internal fixation in the PLFP (plate combined with fracture local fixation) group, that is, on the basis of internal fixation in SPF (simple plate fixation) group of the first half of the picture, additional vertical fixation is performed at the fracture end.

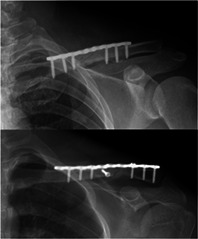

## Introduction

Clavicle fractures account for approximately 2%–5% and 10%–15% of all fractures in adults and children, respectively,^1^ and nearly 80% of clavicle fractures occur in the middle and outer third segments.^2^ They are divided into three types based on radiological review of the anatomical site and the extent of displacement, comminution, and articular extension according to Robinson.^3^ Several studies have reported an improved fracture union rate with surgical treatment of the displaced midshaft and distal clavicle fractures.^4^, ^5^ The displacement of the fracture end of the middle clavicle can affect the movement of the scapula.^6^ The benefits of clavicle fracture surgical treatment must be weighed against the high risk of developing postoperative surgical complications.^7^

Plate and screw fixation is a good option with few complications for simple fractures.^8^ Most treatments of clavicle fractures with plate fixation have a good prognosis.^9^, ^10^, ^11^ Orthopedic and internal fixation can be performed even if the treatment of choice is the conservative treatment of midclavicular fracture malunion.^12^

Usually, after fracture nonunion, complex and expensive treatment is required with multiple surgical procedures, prolonged hospital stay, pain, and functional and psychosocial disability. The ability to promptly identify patients at high risk of nonunion may allow early appropriate targeted treatment intervention leading to a successful outcome. The causes of fracture nonunion and internal fixation loosening failure after clavicular fracture surgery may be closely related to the fracture's morphology and complexity, degree of osteoporosis, and method of internal fixation, rather than just local soft tissue conditions, systemic factors (such as diabetes), infection, allergy, and the stiffness of the internal fixation.^13^ As some scholars believe, the manner of internal fixation is highly correlated with the incidence of clavicular fracture nonunion.^14^ Nicholson *et al*. conducted a 10‐year follow‐up clinical retrospective study of internal fixation of middle clavicle fractures. The results showed that delayed clavicle fixation for more than 3 months was positively correlated with an increased risk of surgical complications and revision surgery.^15^ A meta‐analysis showed that there was no significant difference in the rates of union, nonunion, malunion, and implant failure between the upper and front internal fixation plates for middle clavicle fractures.^16^

The question raised is whether the fracture end needs local vertical fixation in addition to plate fixation. We followed up patients with two different internal fixation methods for a long time, and conducted a follow‐up study focusing on fracture healing, shoulder joint function score, and complications of the two groups of patients. The middle clavicle oblique fracture was only fixed with a plate, and the participants that underwent this procedure comprised the control group. In addition to plate fixation, local vertical fixation, such as Kirschner wire or cortical bone screw or combined internal fixation, was performed at the end of the oblique fracture, and the participants that underwent this procedure comprised the experimental group. Based on this question, we compared the advantages and disadvantages of these two methods and tried to examine the following issues: (i) to judge which internal fixation method is better in the treatment of oblique fracture of the middle clavicle, and clarify whether local fixation is required for fracture end as well as a steel plate; (ii) to explore the recent advances in internal fixation of middle clavicular fracture at home and abroad, and put forward the influence of different internal fixation methods on the curative effect of middle clavicular fracture; and (iii) to analyze the reasons for the differences between the two internal fixation effects using the functional score, complications, and imaging data.

## Materials and Methods

This study was approved by the Clinical ethics committee of Nanjing Medical University (2020CZEY01205). Before surgery, written informed consent was obtained from all participants to allow their anonymized clinical data to be analyzed and published for research purposes.

### Patients

We included patients with: (i) confirmed clavicle Robinson types 2A1 and 2A2 fractures in the middle of the clavicle who underwent open reduction and internal fixation of clavicle fractures through a transverse incision in the anterior clavicle between January 2015 and February 2020; (ii) those who only received plate internal fixation or plate + fracture vertical internal fixation; and (iii) those with a follow‐up period of more than 1 year.

The exclusion criteria were: (i) open fracture; (ii) pathological fracture; (iii) rotator cuff tear leading to ipsilateral shoulder joint dysfunction; (iv) cases combined with acromioclavicular joint dislocation; (v) presence of multiple fractures; and (vi) refusal to undergo plate fixation.

In total, 272 patients were screened for eligibility. This retrospective cohort study enrolled patients with fractures in the middle of the clavicle treated at a third‐grade first‐class hospital. Data were obtained from the hospital information management system.

### Grouping

We carefully explained the possible surgical methods and postoperative precautions to each patient, and answered all their questions. We explained to the patients that both procedures (simple plate fixation [SPF] and plate combined with fracture local fixation [PLFP]) can be successfully performed, but there are differences between them. We emphasized the following aspects: (i) the cost of internal fixation materials in the simple steel plate group is lower; (ii) SPF is more reliable because of the lack of big data and sufficient evidence; (iii) more soft tissue around the fracture may be peeled off in the PLFP group because of the different fracture line direction; (iv) if the patients agree with either method, we will choose the internal fixation according to random grouping arrangements generated by a computer; and (v) patients were advised to perform appropriate functional exercise early after the operation, but avoid lifting heavy objects or performing strenuous exercise involving the affected limbs within 3 months of operation.

### Intervention

All patients in both groups were randomly assigned to three medical groups in the same department. Each medical group was composed of two orthopedic professionals (one senior orthopedist and one senior attending surgeon or one surgical resident). The senior orthopedic surgeons of all medical groups had more than 10 years of experience in fracture surgery, and at least 30 clavicle fracture surgeries were performed every year.

**Fig. 1:**
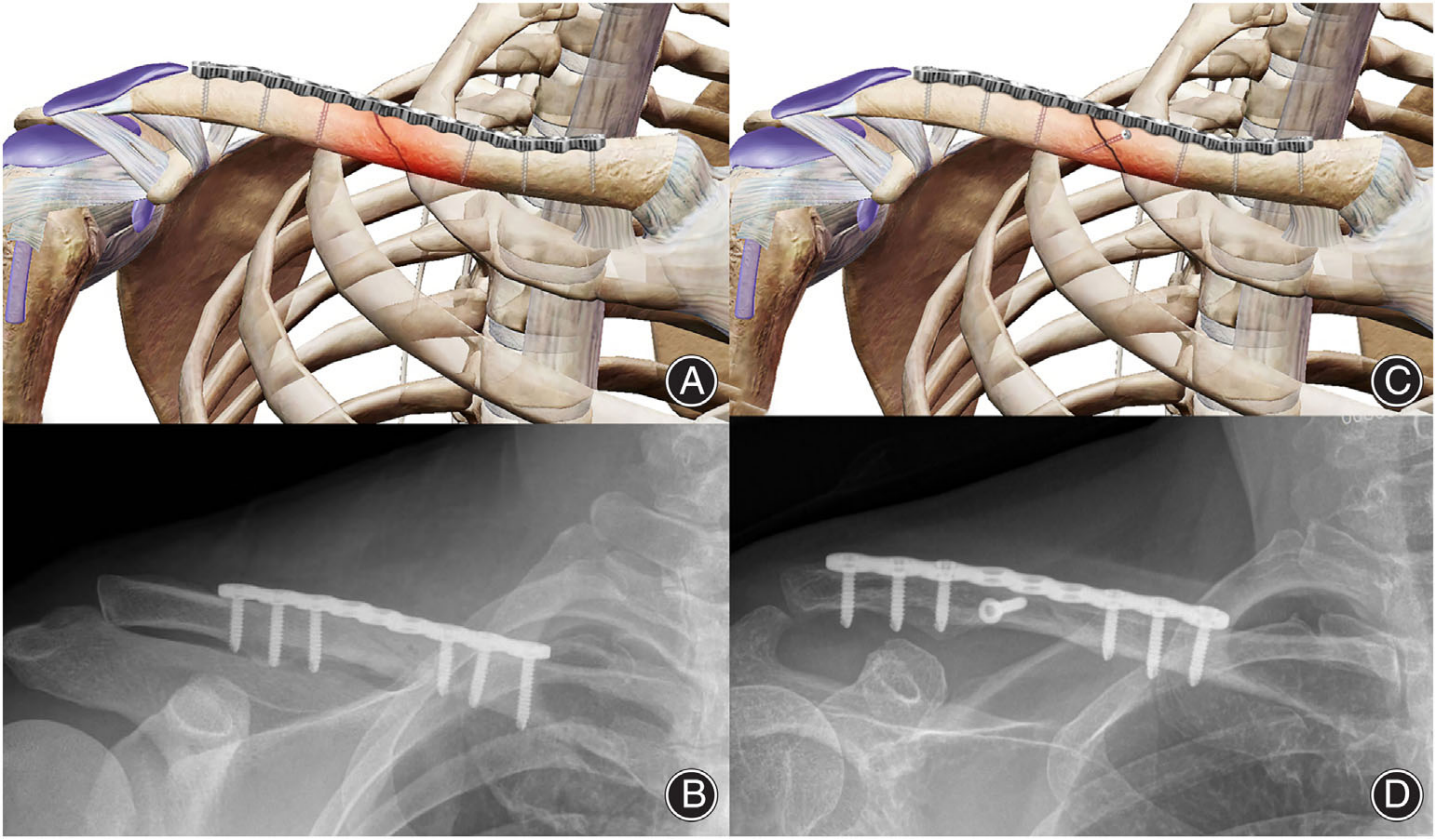
Two kinds of internal fixation and intraoperative X‐ray images. (A) A schematic diagram of plate fixation in the SPF group. There was no external fixation at the fracture end, and the screws on the plate were evenly distributed at both ends of the fracture line. (B) An intraoperative X‐ray image of the clavicle in the SPF group. (C) A schematic diagram of internal fixation in the PLFP group. On the basis of internal fixation in the SPF group, additional vertical fixation was performed at the fracture end. (D) An intraoperative X‐ray image of the PLFP group. Abbreviations: PLFP, plate combined with fracture local fixation; SPF, simple plate fixation

In the SPF group, reconstruction plates or bridging plates with the same biomechanical strength were used only on both sides of the oblique clavicle fracture ends, and the broken ends were not directly fixed vertically (Fig. Fig. 1). However, the fracture ends were cleaned and aligned locally. After the internal fixation device was placed, the fracture ends were tested to ensure that there was no significant loosening or displacement. The method of plate combination with the vertical fixation of the fracture end was mainly based on the simple steel plate group with additional internal fixation at the fracture end (Fig. Fig. 1). The selection of the internal fixator for the vertical fixation of the fracture end was mainly the same material screw or Kirschner wire fixation, and the fixation angle was perpendicular to the fracture line.

In the SPF group, patients were laid in the supine position. After providing cervical plexus anesthesia, the patient's shoulders were raised, and the conventional surgical field was disinfected and covered with a towel. Taking the fracture as the center, an anterior transverse incision of the clavicle, approximately 7–9 cm in length, was made. The skin, subcutaneous tissue, and platysma muscle were cut in turn. The periosteum was cut parallel to the clavicle. The clavicle head and trapezius muscle of the sternocleidomastoid muscle were stripped from the upper edge of the clavicle under the periosteum. Subsequently, the pectoralis major and deltoid muscles were stripped along the lower edge to expose the fracture end. A screw fixation at both ends across the fracture line was made to explore and clean the broken end, relieve local nerve entrapment, release the incarcerated soft tissue, scrape off the blood clot, reduce the fracture, fix the incision with a temporary Kirschner wire, and select the anatomical plate or bridge plate of the appropriate length and type (Fig. Fig. 1).

The choice and mode of incision was similar in both groups. Especially, in the PLFP group, the vertical fixation of the bone fracture was performed before plate fixation (Fig. Fig. 1). The internal fixation perpendicular to the fracture end should be kept at a certain distance from the plate to prevent interaction between them.

### Outcome Measures

#### Patient Characteristics

We evaluated the following indices in both groups: operative time, intraoperative blood loss, postoperative fracture healing time, cases of X‐ray fracture deformity, subjective satisfaction survey, and shoulder function. During follow‐up examinations, malunion was considered when radiographic examination showed that the fracture healing end had flexion, length shortening, angulation, rotation, or overlap. Therefore, we observed whether these deformities affected the activity of the shoulder joint. We defined the healing time of the fracture as the time from surgery to the time when the clavicle was locally and percussive pain‐free with the patient being able to maintain a 1 kg weight for 1 min with the upper extremity extended. The imaging index for fracture healing was callus, spanning 50% of the fracture end. We evaluated the postoperative VAS score and shoulder joint function based on the Constant–Murley and DASH scores.

### Postoperative Outcomes

#### Subjective Patient Satisfaction

We conducted a subjective patient satisfaction survey using a questionnaire that included the following items: foreign body sensation, the fracture reduction satisfaction, cost of hospitalization, surgery satisfaction. The highest score was 6 points, and the lowest score was 0.

#### Visual Analogue Scale (VAS)

The VAS is designed to present to the respondent a rating scale with minimum constraints. Respondents mark the location on the 10‐cm line corresponding to the amount of pain they experienced. VAS data of this type were recorded as the length (in mm) from the left of the line (range, 0–10 mm).

#### Constant–Murley Scale

The Constant*–*Murley scale is the shoulder scoring method adopted by the European Society for Shoulder and Elbow Surgery. The parameters of the left and the right shoulder joint were scored separately. Constant shoulder score mainly includes eight aspects related to use of the shoulder in the past 4 weeks. The proportion of subjective and objective components of the constant shoulder scoring system was 35/65 points. The total score is 100 points. A score of 100 indicates that the patient's condition is normal.

#### Disabilities of the Arm, Shoulder and Hand

DASH is a questionnaire developed to evaluate upper limb function from the perspective of patients. The degree of functional impairment of the affected limb was evaluated by self‐assessment. DASH is divided into two parts, including 30 indicators. The original score is converted into a 100‐point scale. The degree of upper limb function limitation is evaluated according to the score of patients, in which 0 and 100 represent normal and extremely limited upper limb function, respectively.

All the aforementioned outcomes were evaluated by the same investigator and the outcomes assessor was unaware of the treatment group.

#### Follow‐Up Period

The follow‐up time points were at 1, 3, 6, 12, 18, and 24 months. During the follow‐up period, if the internal fixation device was removed, patients were followed up for 3 months postoperatively. One month after the first operation of the clavicle fracture, we investigated postoperative subjective satisfaction.

#### Postoperative Complications

The occurrence of postoperative complications was recorded by another investigator, including mild malunion, nonunion, wound infection, and complication rate.

### Statistical Analysis

Statistical analysis was performed using SPSS 22.0 (IBM, Armonk, NY, USA). We used the Shapiro–Wilk test to determine whether continuous data were normally distributed. Age, operative time, intraoperative blood loss, time of fracture healing, malunion cases, and DASH and Constant–Murley scores were found to be normally distributed, and are presented as means± standard deviations. We analyzed continuous data with equal variances and continuous data with unequal variances using the independent‐samples *t*‐test and Welch–Satterthwaite's *t*‐test, respectively. The pre‐operational and post‐operational VAS scores were compared using the paired sample *t*‐test. Categorical data were analyzed using the chi‐squared test. The level of significance was set at *P* < 0.05.

## Results

### Patient Characteristics

**Fig. 2:**
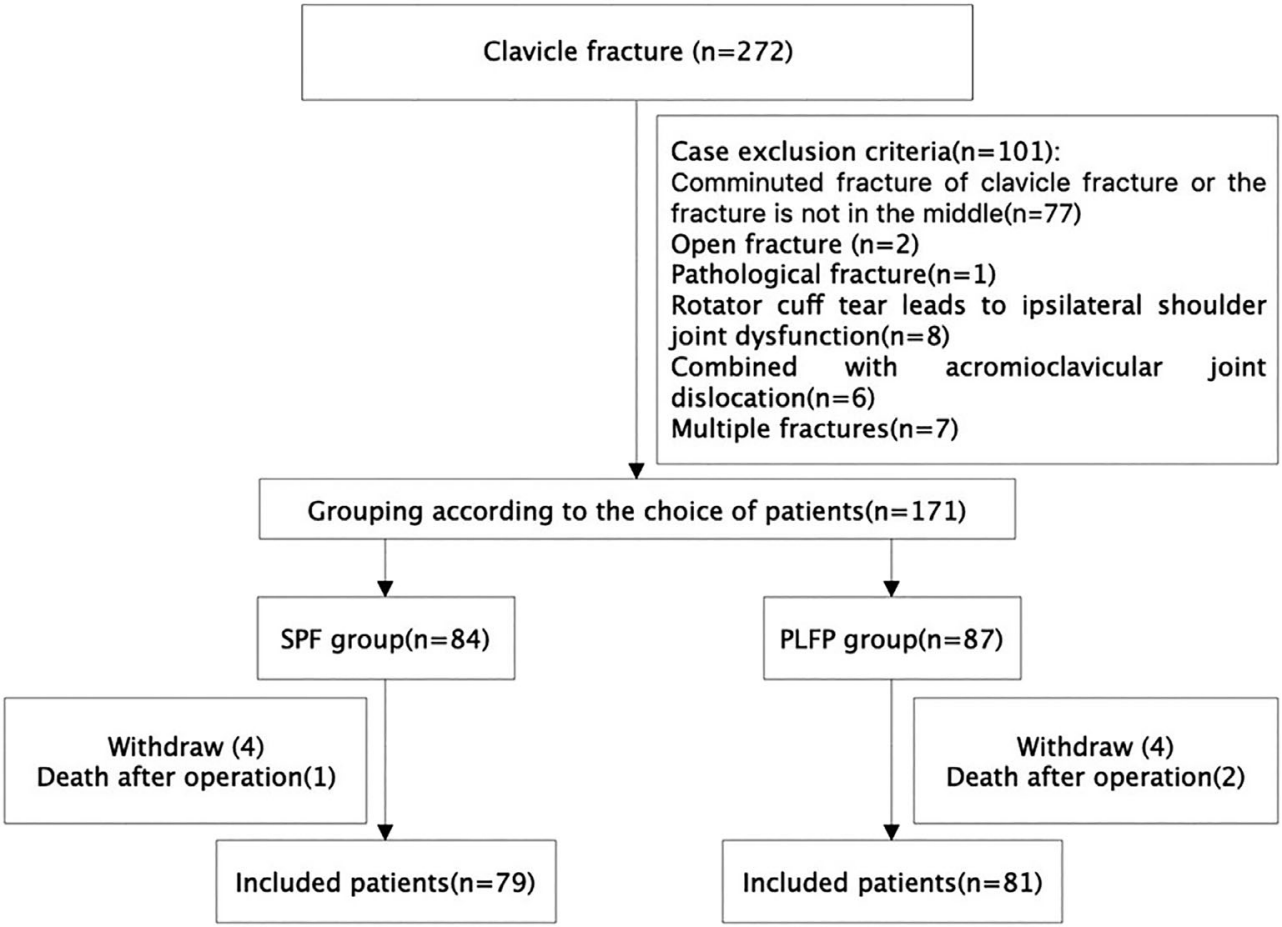
Study flowchart

**Fig. 3:**
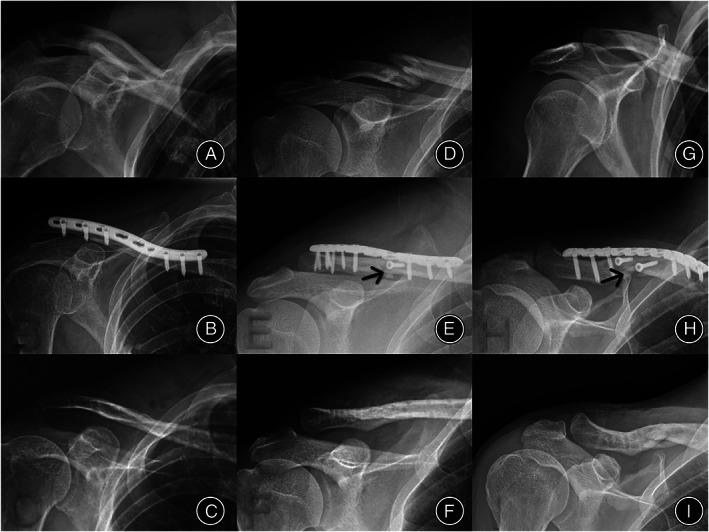
Radiographic findings of the healing process of three cases with different internal fixation methods. (A–C) Images of the SPF group before operation, after operation, and during fracture healing after removal of the internal fixation device, respectively. (D–F) Images of the PLFP group before operation, after operation, and during fracture healing after removal of the internal fixation device, respectively. The black mark in panel E indicates that the fracture end was vertically fixed outside the plate with a screw. (G–I) Images of the PLFP group before operation, after operation, and during fracture healing after removal of the internal fixation device, respectively. The black mark in panel H indicates that the fracture end is vertically fixed outside the plate with two screws. (C, F, and I) The fracture was healed well after the removal of internal fixation devices in both groups. Abbreviations: PLFP, plate combined with fracture local fixation; SPF, simple plate fixation

We screened 272 patients for participation between January 2015 and February 2020. In total, 171 patients were enrolled. Eighty‐four and 87 patients were included in the SPF and PLFP groups, respectively (Fig. 2). We included 160 patients with a sufficient follow‐up period in the final analysis: 79 in the SPF group (follow‐up duration: 16.24 ± 3.94 months) and 81 in the PLFP group (follow‐up duration: 16.15 ± 3.43 months) (Fig. 2). Age, sex, body mass index, follow‐up duration, fracture classification, and cause of injury were not significantly different between the two groups (Table 1). There were no significant differences between the two groups in terms of intraoperative blood loss (*P* < 0.05) (Table 2). Compared with the SPF group, the operation time was longer in the PLFP group (the average operation time of the PLFP group was 1.14 times longer than that of the SPF group), but the time of fracture healing was shorter in the PLFP group (the average fracture healing time was 1.10 times longer in the SPF group than in the PLFP group) (Table 2). According to the different internal fixation methods, the typical cases are presented in Figure 3 from the imaging data before and during the operation and after the fracture healing.

**TABLE 1:** Characteristics of the patients

Demographics	SPF group (*n* = 79)	PLFP group (*n* = 81)	*t*‐ value	*P*‐value
Age (years)	46.37 ± 14.54	48.42 ± 12.55	−0.96	0.34
Sex: male (*n*, %)	43, 54.43	36, 44.44	1.59	0.11
Body mass index (kg/m^2^)	23.65 ± 3.22	23.64 ± 3.23	0.03	0.98
Follow‐up (months)	16.24 ± 3.94	16.15 ± 3.43	0.16	0.87
Fracture (Robinson type, *n*, %)			0.61	0.54
2A1	4, 5.06	6, 7.41		
2A2	75, 94.94	75, 92.59		
Injury causes (*n*, %)			0.91	0.37.
Car crash injury	17, 21.52	22, 27.16		
Electric vehicle injury	36, 45.56	37, 45.68		
Walking, slipping and falling injury	18, 22.78	15, 18.52		
Others	8, 10.14	7, 8.64		

*Note*: Data are presented as *n* or mean ± standard deviation.

**TABLE 2:** Surgical results of the patients

Surgical results	SPF group (*n* = 79)	PLFP group (*n* = 81)	*t*‐ value	*P*‐value
Intraoperative blood loss (ml)	63.29 ± 31.63	67.22 ± 22.62	−0.91	0.366
Surgical time (min)	57.61 ± 15.92	65.48 ± 16.48	−3.07	0.002^a^
Fracture healing time (month)	4.87 ± 1.60	4.41 ± 0.99	2.22	0.028^a^
Constant–Murley score	86.18 ± 6.10	87.62 ± 3.95	−1.78	0.077
DASH score	7.03 ± 2.42	6.73 ± 2.00	0.85	0.399
Subjective satisfaction	4.13 ± 1.91	4.27 ± 1.14	−0.79	0.433
Complications (*n*, %)	7, 8.86	5, 6.17	0.69	0.49
Mild deformity healing	2	1	0.60	0.55
Fracture nonunion	2	0	1.44	0.16
Postoperative superficial tissue infection	3	4	0.04	0.97

1 *Notes*: Data are presented as *n* or mean ± standard deviation.

^a^ The differences in Surgical time and Fracture healing time between the two groups were determined using the Satterthwaite *t*'‐test.

### Postoperative Outcomes

#### Subjective Patient Satisfaction

The satisfaction scores of Groups SPF and PLFP were 4.37 ± 0.72 and 4.46 ± 0.59 points, respectively. There were no significant differences between the two groups in the Subjective Patient Satisfaction scores (*P* = 0.39) (Table 2).

#### VAS Score

The postoperative VAS score of the SPF group (3.16 ± 0.81) was significantly improved compared to the preoperative VAS score (7.72 ± 0.68) (*P* < 0.05). Similarly, the postoperative VAS score of the PLFP group (3.30 ± 0.72) was significantly improved compared to the preoperative VAS score (7.65 ± 0.62) (*P* < 0.05). There was no significant difference between the two groups (*P >* 0.05).

#### Constant–Murley Scale

There were no significant differences between the two groups in terms of the Constant–Murley scores (*P* = 0.07). The specific scores of the SPF and PLFP groups were 86.18 ± 6.10 and 87.62 ± 3.95, respectively (Table 2).

#### DASH Score

Compared with the SPF group, the DASH score of the PLFP group did not show significant worsening (*P* = 0.40). The DASH scores were 7.03 ± 2.42 and 6.73 ± 2.00 in the SPF and PLFP groups, respectively (Table 2).

#### Postoperative Complications

There were 79 patients in the SPF group, and seven patients had complications (complication rate, 8.86%). Complications included mild malunion (*n* = 2), nonunion (*n* = 2), and wound infection (*n* = 3). The functional scores of five patients were lower than those of other patients.

There were 81 patients in the PLFP group, and five of them had complications (complication rate, 6.17%). Complications included mild malunion (*n* = 1), nonunion (*n* = 0), and superficial wound infection (*n* = 4). There was no significant difference between the two groups in complication rates (*P* = 0.713).

## Discussion

### Clinical Results of the SPF and PLFP Groups

A plate combined with the vertical fixation of the fracture end may be a potential method to treat oblique fractures of the middle clavicle. Therefore, our aim was to compare the clinical efficacy of performing plate fixation with that using a plate combined with vertical fixation outside the plate in the treatment of oblique fracture of the middle clavicle. The fracture healing time was shorter in the PLFP group than in the SPF group. However, the operation duration was longer in the PLFP than in the SPF group. These two treatments have equivalent therapeutic effects on shoulder joint function recovery.

### Therapeutic Selection of Middle Clavicle Fracture

The tendency of choosing surgery rather than conservative treatment for clavicular fracture is stronger. For displaced clavicle fractures, conservative treatment may lead to the malunion of the fracture site and a high rate of nonunion.^17^, ^18^ It has been reported that the symptomatic malunion of middle clavicle fractures after surgical treatment is significantly lower than that after conservative treatment.^19^, ^20^ The abnormal healing of the clavicle will lead to the loss of length, which may cause an imbalance of shoulder muscle force and affect the activity of the shoulder joint.^21^ When there are surgical indications, open reduction and internal fixation are mainly used to treat clavicle fracture, and the current method mainly includes intramedullary fixation (IM) and plate fixation. However, IM and plate fixation are associated with some difficulties in the treatment for middle clavicle fractures. A meta‐analysis of the study indicated that plate fixation seemed to form a more robust construct than intramedullary fixation in terms of stiffness and failure loading;^22^ however, the remaining clavicle was stronger after the removal of the intramedullary device than after the removal of the plate. Each type of internal fixation devices has advantages and disadvantages in terms of biomechanics. Chan *et al*. conducted a long‐term follow‐up study of plate fixation and intramedullary fixation for two‐part and multi‐segmental displaced middle clavicle fractures, which showed that IM and plate fixation had the same nonunion rate and lower complication and revision rates, but the plate had a lower nonunion rate for multi‐segmental displaced mid clavicle fractures.^23^ However, some scholars believe that IM is better than plate fixation. A previous meta‐analysis revealed that IM significantly shortened the operation duration and the incidence of non‐surgical complications, especially the infection rate.^24^ Simultaneously, Gao *et al*. also obtained the same results in the clinical comparative study of IM and plate fixation for middle clavicle fractures.^25^ IM has the potential advantages of a smaller incision, decreased dissection, and soft‐tissue exposure. IM substitutes, such as Kirschner wires, titanium elastic nails, and cannulated screws, are often used to treat clavicle fractures.^26^ Similarly, steel plates are considered a “gold standard” for midclavicular fractures.^27^ When a clavicle fracture is fixed with a plate, there are different opinions on the optimal position of the plate, type of plate, and type of screw device. A part of the reason for IM being preferred over plate fixation may be that load conditions used in different studies are different, and the anatomical structure of the shoulder is complex; therefore, it is difficult to conduct actual experimental tests.^28^ Marie used a finite element method to simulate the stress of the clavicle plate and bone. By looking at the clavicle fixation from a mechanical point of view, the results indicate that it is a major benefit to use a lag screw to fixate the fracture.^28^ In this clinical study, two groups of patients with a vertical fixation of the local fracture line were followed up for a long time, and the results were different from those of Marie's study. The results of this study suggest that the method of a plate combined with the vertical fixation of the fracture end does not have an additional benefit.

### Analysis of Differences in the Baseline Data between the Two Groups

In our study, there was no significant difference in blood loss or postoperative complications (superficial infection, malunion, and nonunion) between the PLFP and SPF groups. However, the fracture healing time was lower in the PLFP than in the SPF group. There was no significant difference in the operative approach and perioperative management between the two groups. This article did not include and study the impact of multiple fractures, such as those combined with other high‐energy vitamin fractures, and pathological fractures on both groups. The main difference between the two groups was the difference in internal fixation methods. A retrospective analysis of the two groups of patients with mild malunion in the follow‐up process found that the fracture ends of the patients in both groups were rarely displaced at 1 month postoperatively. After a detailed inquiry of the rehabilitation process of the patients, we found that because of the fear of the fracture itself and early pain of the wound, early shoulder functional exercise was limited. However, with the recovery of soft tissue and the relief of pain symptoms, patients gradually returned to their daily life and work, and the amount and load of the shoulder gradually increased, which then, produced slight angular displacement of the fracture ends that may eventually lead to the occurrence of mild malunion. Zhang *et al*. conducted a finite element analysis of single and double plate fixations for middle clavicle fractures, and also mentioned that single‐plate fixation patients should avoid load‐bearing and excessive shoulder joint movement so as to prevent the failure of fixation and the displacement of the fracture end.^29^ There was no significant difference in the incidence of malunion between the two groups in our study. In addition, previous studies have shown that infection, nonunion or fracture of the plate may occur in all cases with plate fixation.^30^ Although the total number of cases was lower in the SPF than in the PLFP group, cases with mild malunion were more in the PLFP group. This also suggests that if the sample size increased, the probability of malunion in the SPF group may be higher.

### Advantages and Disadvantages of Intramedullary Fixation and Plate Fixation

Another issue that requires consideration is whether only intramedullary device substitutes (i.e., Kirschner wire and screw) can be used to treat middle clavicle fractures. Although intramedullary fixation has a lower infection rate, as well as lower complication and revision rates than plate fixation,^24^, ^25^ there is a lack of large sample data and high‐quality randomized controlled studies in the treatment of clavicle fractures with intramedullary replacement devices instead of plate fixation. Moreover, some case studies have shown that the use of only Kirschner wire fixation in the treatment of the clavicle fracture has a risk of fracture displacement,^31^ even in spinal cord injury cases.^32^ Simply using screws to fix the fracture end was rarely reported. Jin *et al*. achieved good results in the treatment of the clavicle fracture by closed reduction cannulated screws, but the number of cases was only 17, and one case presented screw loosening.^33^ These findings suggest that the treatment of clavicle fractures with only intramedullary device substitutes may not be reliable. In this study, in addition to plate fixation, the PLFP group also used intramedullary device substitutes (Kirschner wire or/and screw) to vertically fix the broken end of the fracture. Results showed that the PLFP group had low displacement and malunion rates, as well as low delayed union and nonunion rates, indicating that it could help patients to perform early shoulder rehabilitation exercises.

### Insights of Different Internal Fixation Methods for Oblique Fracture of the Midshaft Clavicle

In summary, the curative effect of plate fixation or plate combined with a vertical fixation device in the treatment of middle clavicle oblique fractures was similar. The two fixation methods presented their own advantages and disadvantages. By performing SPF, the cost can be reduced, the operation time can be shortened, and the degree of soft tissue peeling at the broken end of the fracture can be reduced. The healing time of fractures was longer in the SPF than in the PLFP group. However, it will not significantly affect the final clinical outcome. In the PLFP group, it is helpful for the early healing of fractures with higher costs and longer operation duration, but it may be more helpful for early functional exercises of patients.

### Limitations

This study had some limitations. All samples were obtained from the same hospital, and the sample size was limited. Although the surgical approach was the same and the chief surgeon was an experienced chief physician in the same department, all operations were not performed by the same team. Finally, in the grouping, the factors of the patients' choices were considered. Thus, the selection of grouping was not randomized and blinded, which may have led to some deviation. To confirm our findings, it is necessary to conduct more prospective randomized controlled studies.

### Conclusion

For the oblique fracture of the middle clavicle, both SPF and a. PLFP can achieve good shoulder joint function recovery. Considering the possibility of fracture deformity and nonunion, patients undergoing SPF should avoid early weight‐bearing and performing excessive shoulder joint activity. If economic conditions allow, it is recommended to choose the method of a plate combined with the vertical fixation of the fracture end to achieve faster fracture healing.

## Conflict of Interest

The authors have no conflicts of interest to declare.

## Footnotes

1 Abbreviation: DASH, disabilities of the arm, shoulder and hand.
